# Comparing occasional and persistent frequent attenders in occupational health primary care – a longitudinal study

**DOI:** 10.1186/s12889-018-6217-8

**Published:** 2018-11-26

**Authors:** Tiia Reho, Salla Atkins, Nina Talola, Markku Sumanen, Mervi Viljamaa, Jukka Uitti

**Affiliations:** 1 Faculty of Medicine and Life Sciences, University of Tampere, PB 100, FI-33014 Tampere, Finland; 2 Pihlajalinna Työterveys, Tampere, Finland; 3 Department of Public Health Sciences, Karolinska Institutet, Stockholm, Sweden; 4 Finnish Institute of Occupational Health, Tampere, Finland; 5 Clinic of Occupational Medicine, Tampere University Hospital, Tampere, Finland

**Keywords:** Frequent attender, High user, High utilizer, Occupational health services, Persistent frequent attendance, Primary health care, Health care utilization, Longitudinal studies

## Abstract

**Background:**

The aim of the study was to compare occasional and persistent frequent attenders in occupational health (OH) primary care and to identify the diagnoses associated with persisting frequent attendance.

**Methods:**

This is a longitudinal study using electronic medical record data from 2014 to 2016 from an OH service provider. Frequent attenders were defined as patients in the top decile of annual visits to healthcare professionals (frequent attender 10%, FA10). FA10 were categorized to three groups according to the persistence of frequent attendance (1-year-FA, 2 year-FA, and persistent-FA = frequent attenders in all three years). This was used as the dependent variable. We used patient sex, age, employer size, industry and distribution of visits and diagnostic codes to characterize the different frequent attender groups.

**Results:**

In total, 66,831 patients were included, of which 592 persistent frequent attenders (0.9% of the study population) consulted the OH unit on average 13 times a year. They made altogether 23,797 visits during the study years. The proportion of women and employees of medium and large employers increased among persistent-FAs when compared to the other groups. Multinomial logistic regression accentuated musculoskeletal disorders and to a lesser extent diseases of the respiratory and nervous system and mental disorders. One in five FA becomes a persistent-FA.

**Conclusions:**

Our results indicate that in the context of a working population the association of musculoskeletal disorders and persistent frequent attendance is emphasized. Persistent frequent attenders also create a substantial demand on physician resources. When planning interventions aimed at working age frequent attenders, subgroups suffering from musculoskeletal disorders should be identified as they are associated with persisting frequent attendance.

## Background

Frequent attenders demand a substantial portion of physician’s time and consume a considerable share of health care resources [1–3]. Some patients consult their physician repeatedly for a short period and return to an irregular pattern of attendance after some time [3, 4]. Another group of patients, often referred to as persistent frequent attenders, visit health care providers frequently one year after another [3, 5]. Though studies on persistent frequent attendance are sparse, and concentrate on a general practice setting, it appears that a combination of somatic, psychological and psychiatric, and social factors lead to persistent frequent attendance [4–6]. In order to purposefully direct resources and to provide adequate treatment and rehabilitation, we need to be able to recognize individuals at risk of continuous high use of services with the routine data available during consultations. In addition, the differentiation of occasional and persistent frequent attenders could be useful for service planning as studies suggest that persistent FA’s consume an even larger proportion of physicians time yearly than occasional FA’s, and present more social problems and higher morbidity [3, 5] than occasional FA’s.

Previous research suggests that frequent attenders suffer from multimorbidity [6, 7] and low quality of life [8]. Studies also indicate that unemployment is associated with frequent attendance especially among men [9, 10] but few studies thus far have concentrated on frequent attendance among the working population [11]. Studies conducted in general practice or secondary care setting do not address the demands of the working life. Given that work has beneficial effects on health [12] but also places demands on work ability, the working population should be examined also separately. Studying the working population could yield different results possibly emphasizing illnesses that restrict work ability. Finnish occupational health (OH) primary care is an appropriate environment to study frequent attenders in working population, as it covers 90% of the employees [13] and maintains comprehensive health records.

Visits to occupational health services (OHS) primary care are associated with chronic illnesses affecting work ability and work related symptoms [14]. Chronic health issues are also associated with lower productivity at work [15] and lowered work ability, which supports their being treated and managed in OHS. The most common work-related visits to the OH physician are musculoskeletal and mental disorders [16], which are both also leading causes of disability in Finland [17] and linked to frequent attendance in general practice setting and OH primary care [11, 18, 19]. This suggests that frequent attenders in OH primary care might be a vulnerable group of patients demanding careful assessment of work ability, work relatedness and follow up. Given the complexity of frequent attenders’ conditions and the resource demand they create, it is crucial that their conditions are identified as early as possible. It is also pertinent to differentiate characteristics and factors associated with occasional and persistent frequent attendance to determine which groups need OH interventions. Identifying the risk groups would allow targeted OH examinations, where health plans and necessary rehabilitative measures and work place interventions can be planned to prevent disability [20].

We aimed to compare occasional and persistent frequent attenders and to define factors associated with persistent frequent attendance in OH primary care.

## Material and methods

### Study setting and design

Primary health care services in Finland are organized in three parallel structures: municipal, private and occupational health care (OH). Preventive occupational health services are mandated by law and employers arrange these services for employees. In addition most employers arrange for the same health care provider that provides legislative services also to provide primary care services for employees – OH primary care covers approximately 90% of the working force [13].

This is a longitudinal retrospective study using routine medical record data from a large private OHS provider Pihlajalinna Työterveys which has 40 OH units around the nation. A longitudinal study design was chosen to analyze predictive factors associated with persisting frequent attendance. Pihlajalinna Työterveys’ clients represent the working population of Finland including companies from a wide range of industries and rural as well as urban areas. In OHS primary care patients can use services of different health care professionals who are usually specialized in occupational health: physicians, nurses, physiotherapist and psychologists. A referral from a nurse or physician is required for a physiotherapist or psychologist consultation and physicians can consult other medical specialists. In Finland occupational health negotiations (referred to as OH collaborative negotiation) [21] are held confidentially between the occupational health physician, employee and employer whenever concerns are raised on the individuals work ability.

### Data collection

Our data consisted of routine information, including diagnostic codes, entered during all visits to healthcare professionals in 2014–2016. The data also included background data, such as age and sex of the employee and employer’s size and industry. Information on OH collaborative negotiations held was also obtained. The data were collected by Pihlajalinna and sent in pseudonymized form to the University of Tampere. Pseudonymization was carried out by Pihlajalinna Työterveys and University of Tampere received the data including only ID-number than cannot be associated with a single patient. The corresponding social security number and ID-list was kept by Pihlajalinna. Based on Finnish legislation (Personal Data Act, Finland, 22.4.1999) individual consent is unnecessary since no individual could be identified due to the size of the study population.

Our initial data comprised 78,507 patients. The study material was limited to employees aged 18–68 years who had visited the OHS primary care face-to-face at least once during the study years. All general and mandatory health check-ups and contacts not conducted face-to-face (prescription renewals, telephone calls etc.) were excluded based on invoice codes. General and mandatory (occupational) health check-ups were excluded as they are not initiated by the patient nor are they necessarily illness related. After these exclusions our study comprised 66,831 patients. Diagnostic codes (ICD-10) registered for each physician visit were collected and the first (i.e. the main) diagnosis was used in the analysis.

### Statistical analysis

**Fig. 1:**
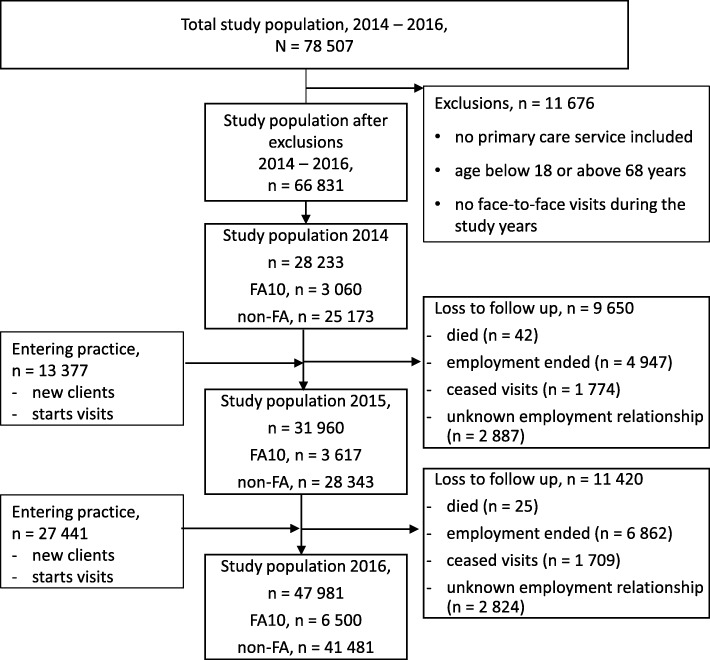
Flow diagram of patient categorization into FA10 and non-FA. FA10 = the top decile of attenders (frequent attender 10%, FA10). non-FA = patients that were never in the top decile were considered as a reference group, non-frequent attenders

Frequent attendance was defined as top decile of attenders [3, 22]. Visits to physicians, nurses, physiotherapist and psychologists were used to determine the top decile of attenders (frequent attender 10%, FA). Patients were then categorized into four groups for analysis. Those patients that were in the top decile of attenders in one of the study years (2014, 2015 or 2016) were named 1-year-FA (1yFA). The patients that were in the top decile in any two study years were named 2-year-FA (2yFA). Those patients that were in the top decile in all three study years were considered persistent frequent attenders (pFA). Patients that were never in the top decile were considered as a reference group, non-frequent attenders (non-FA). A flow diagram of patient categorization and loss to follow up is shown in diagram 1 (Fig.1).

The study population was divided into four age groups (18–34, 35–44, 45–54, 55–68) and further by sex. In further analysis no age stratification was done since the whole study population consists of working age population. Employers were categorized according to number of employees (micro 1–10, small 11–50, medium 51–250 and large > 251 employees). The employer industry was classified according to Statistics Finland (TOL2008/Nace Rev. 2) and the 10 largest industries were analyzed separately and the 10 smaller industries were combined as one group (others). Diagnoses registered at the physician visits were categorized according to the chapter headings of ICD-10. ICD-10 subgroups were defined in more detail based on previous literature [3, 18, 19] and to examine the largest diagnostic groups more closely [11].

Descriptive statistics were used to examine demographic data, OH collaborative negotiation and background data including employer size and industry of the frequent attenders groups (1yFA, 2yFA, pFA or non-FA). Differences between the groups in characteristics were analyzed using Pearson’s chi-square. One-way ANOVA tests was used to analyze the number of visits to different health care professionals as a whole and the distribution of visits between different professional groups. Kruskal-Wallis –test was used to analyze differences between the groups in the number of diagnoses. In multinomial logistic regression the outcome variable was categorized into four: non-FA, 1yFA, 2yFA and pFA. We used the non-FA group as a reference group. The analysis was adjusted by sex, age, employer’s field of industry and size. Odds ratios (OR) with 95% confidence intervals (95% CI) were determined for each factor (professionals visited, diagnosis). *P* values under 0.05 were considered statistically significant. Statistical analyses were performed with IBM SPSS Statistics version 23 (IBM Corp., Armonk, NY, USA) software by NT.

## Results

The study population after exclusions comprised 66,831 patients (2014–2016). When divided into four categories 592 (0.9%) patients were pFAs, 1603 (2.4%) 2yFAs, 6528 (9.8%) 1yFAs and 58,108 (86.9%) non-FAs. Proportionally more women (50% of 1yFA, 53% of 2yFA and 56% of pFA) than men were frequent attenders (and the proportion of women increased in 2yFAs and pFAs). Frequent attenders were predominantly employed in medium and large companies (Table 1). The three largest industries employing frequent attenders were manufacturing, public administration and human health and social work (data not shown). The use of other professionals besides physicians increased as frequent attendance continued. 2yFAs and pFAs consult with a psychologists, physiotherapists and specialists more often than non-FAs and 1yFAs do. In addition, the likelihood of occupational health negotiation increased as frequent attendance persisted. See Table 1 for further characteristics.

**Table 1:** Study population 2014–2016, characteristics of 1-year-FA, 2-year-FA, pFA and non-FA (*n* = 66,831)

Characteristics	1-year-FA 2014–2016		2-year-FA 2014–2016		pFA 2014–2016		non-FA 2014–2016		*p* value
	*n*	(%)	*n*	(%)	*n*	(%)	*n*	(%)	
	6528	(10)	1603	(2)	592	(1)	58,108	(87)	
Sex									< 0.001
Male	3270	(50)	754	(47)	262	(44)	33,236	(57)	
Female	3258	(50)	849	(53)	330	(56)	24,872	(43)	
Age									< 0.001
18–34	1661	(25)	354	(22)	128	(21)	19,630	(34)	
35–44	1641	(25)	413	(26)	147	(25)	13,648	(23)	
45–54	1889	(29)	473	(30)	187	(32)	14,351	(25)	
55–68	1337	(21)	363	(22)	130	(22)	10,479	(18)	
Company size									< 0.001
0–10	507	(8)	77	(5)	19	(3)	8544	(15)	
11–50	1601	(25)	350	(22)	129	(22)	16,036	(28)	
51–250	1767	(27)	513	(32)	195	(32)	14,165	(24)	
> 250	2287	(35)	663	(41)	249	(42)	16,451	(28)	
Missing	2	(0)					16	(0)	
Specialist consultation									< 0.001
No	4677	(72)	894	(56)	244	(41)	51,622	(89)	
Yes	1851	(28)	709	(44)	348	(59)	6486	(11)	
Professionals visited									< 0.001
Physician	6513	(100)	1603	(100)	592	(100)	53,945	(93)	
Nurse	4119	(63)	1192	(74)	460	(78)	18,918	(33)	
Physiotherapist	2932	(45)	1023	(64)	425	(72)	7910	(14)	
Psychologist	1174	(18)	467	(29)	196	(33)	1966	(3)	
OH collaborative negotiation (2014–2015)									< 0.001
No	6309	(97)	1424	(89)	453	(77)	57,490	(99)	
Yes	219	(3)	179	(11)	139	(23)	618	(1)	

FA status was defined as the top decile of attenders (frequent attender 10%, FA10)

1-year-FA = Patients that were in the top decile of attenders in one of the study years (2014, 2015 or 2016)

2-year-FA = Patients that were in the top decile in any two study years (2014, 2015 or 2016)

pFA = Patients that were in the top decile in all three study years (2014, 2015 and 2016)

non-FA = Patients that were never in the top decile were considered as a reference group, non-frequent attenders

The average and mean consultation rates can be seen in Table 2. Persistent frequent attenders consult with a healthcare professional yearly over five times more than non-FAs do. The differences between consultation rates were notable in physician consultations but the same trend was seen also with other health care professionals. Over the three study years, pFAs attended their OH primary care unit 40 times on average whereas a non-FA visited on average 4 times. Most of these consultations were doctor’s appointments. Over the three year period physiotherapists were consulted on average 1.3, 2.6, 4.0 and 0.2 times (md 0, 1, 2 and 0) by 1yFA, 2yFA, pFA and non-FA respectively. Over the same period psychologists were consulted on average 1.4 times by pFA and 0.6, 1.3 and 0.08 times (md 0) by 1yFA, 2yFA and non-FA respectively.

**Table 2:** Association between consultation visits and frequent attender status (*n* = 28,233–66,831)

Characteristics	Consultations, all		Physician		Nurse	
	av.	md	av.	md	av.	md
2014 (*n* = 28,233)	***		***		***	
1-year-FA	4.9	4	3.6	3	0.7	0
2-year-FA	7.7	8	5.6	5	1.1	0
pFA	13.2	11	9.6	9	1.8	1
non-FA	2.9	2	2.3	2	0.4	0
2015 (*n* = 31,960)	***		***		***	
1-year-FA	5.7	5	4.1	4	0.8	0
2-year-FA	10.2	9	7.3	7	1.4	1
pFA	14.3	13	10.6	10	1.8	1
non-FA	2.7	2	2.1	2	0.4	0
2016 (*n* = 47,981)	***		***		***	
1-year-FA	7.8	8	5.5	5	1.2	0
2-year-FA	9.4	9	6.7	6	1.2	0
pFA	12.6	11	9.4	8	1.5	1
non-FA	2.4	2	1.9	1	0.4	0
2014–2016 (*n* = 66,831)	***		***		***	
1-year-FA	13.8	13	9.9	9	2.1	1
2-year-FA	26.4	25	19.0	19	3.6	2
pFA	40.0	37	30.0	28	5.1	3
non-FA	4.0	3	3.1	2	0.6	0

One-way ANOVA –test, av. = average, md = median, *p* < 0.001 in all values

FA status was defined as the top decile of attenders (frequent attender 10%, FA10)

1-year-FA = Patients that were in the top decile of attenders in one of the study years (2014, 2015 or 2016)

2-year-FA = Patients that were in the top decile in any two study years (2014, 2015 or 2016)

pFA = Patients that were in the top decile in all three study years (2014, 2015 and 2016)

non-FA = Patients that were never in the top decile were considered as a reference group, non-frequent attenders

Table 3 includes the distribution of diagnoses for 1yFA, 2yFA, pFA and non-FA. When examining the diagnostic codes registered for each physician visit, the most common diagnostic codes for any group were diseases of the respiratory system and of the musculoskeletal system. Diseases of the musculoskeletal system were overrepresented in frequent attender groups and their frequency increased towards persistent frequent attendance. The same trend is visible in all the diagnostic groups and is accentuated also in mental and behavioural disorders, injuries and unclassified symptoms. During the three study years average number of different diagnoses was 4.2 (md 4), 5.8 (md 6), 6.9 (md 7) and 2.0 (md 2) for 1yFA, 2yFA, pFA and non-FA respectively (*p* <  0.001, Kruskal-Wallis -test).

**Table 3:** Patients diagnosed with a disease according to ICD-10 (registered for physician consultations in the study years 2014–2016, *n* = 66,831)

Characteristics	1-year-FA 2014**–**2016		2-year-FA 2014**–**2016		pFA 2014**–**2016		non-FA 2014**–**2016	
	*n*	(%)	*n*	(%)	*n*	(%)	*n*	(%)
	6528	(10)	1603	(2)	592	(1)	58,108	(87)
ICD-10								
J00-J99 Diseases of the respiratory system	4254	(65.2)	1321	(82.4)	536	(90.5)	23,678	(40.7)
M00-M99 Diseases of the musculoskeletal system and connective tissue	4796	(73.5)	1422	(88.7)	559	(94.4)	21,303	(36.7)
R00-R99 Symptoms, signs and abnormal clinical and laboratory findings, not elsewhere classified	2309	(35.4)	857	(53.5)	401	(67.7)	9147	(15.7)
S00-T98 Injury, poisoning and certain other consequences of external causes	2198	(33.7)	792	(49.4)	349	(59.0)	9228	(15.9)
L00-L99 Diseases of the skin and subcutaneous tissue	1335	(20.5)	510	(31.8)	220	(37.2)	5717	(9.8)
F00-F99 Mental and behavioural disorders	1595	(24.4)	609	(38.0)	270	(45.6)	4663	(8.0)
I00-I99 Diseases of the circulatory system	1129	(17.3)	403	(25.1)	168	(28.4)	4902	(8.4)
A00-B99 Certain infectious and parasitic diseases	1102	(16.9)	425	(26.5)	228	(38.5)	4827	(8.3)
H00-H59 Diseases of the eye and adnexa	868	(13.3)	326	(20.3)	163	(27.5)	4056	(7.0)
H60-H95 Diseases of the ear and mastoid process	909	(13.9)	315	(19.7)	153	(25.8)	3687	(6.3)

ICD-10 = International Classification of Diseases

FA status was defined as the top decile of attenders (frequent attender 10%, FA10), *p* < 0.001 in all values

1-year-FA = Patients that were in the top decile of attenders in one of the study years (2014, 2015 or 2016)

2-year-FA = Patients that were in the top decile in any two study years (2014, 2015 or 2016)

pFA = Patients that were in the top decile in all three study years (2014, 2015 and 2016)

non-FA = Patients that were never in the top decile were considered as a reference group, non-frequent attenders

In the table are presented the 10 largest ICD-10 groups

Table 4 shows the adjusted OR for factors associated with frequent attendance of varying lengths. The same ICD-10 categories dominated in all three categories but the proportions differed to some extent. Among pFA diseases of the musculoskeletal and respiratory system had the highest odds, followed by unclassified symptoms (R00-R99). On the other hand among 1yFAs musculoskeletal and mental disorders were the leading diagnoses and diseases of the nervous system had the third highest OR. Among 2yFAs musculoskeletal and respiratory diseases dominated but mental and behavioural disorders were third most common. Diseases of the nervous system and injuries stood out in all three FA categories. When examining the ICD-10 F-codes more closely we noted that for depressive episodes the adjusted OR for pFA was 12.0 (95% CI 9.5–15.2) and for phobic disorders 8.5 (95% CI 6.5–11.0). For illnesses of the back and spine OR for pFA was 13.5 (95% CI 11.3–16.1) and illnesses of the neck, cervical spine and tension headache the OR was 10.47 (95% CI 8.9–12.4). For illnesses of the upper extremities the OR was 8.9 (95% CI 7.5–10.5) and for illnesses of the lower extremities 7.9 (95% CI 6.7–9.4). Again, for pFA the OR for asthma and COPD was 8.3 (95% CI 6.4–10.7) while for acute upper respiratory infections the OR was 13.4 (95% CI 10.7–16.9) (data not shown). We also saw that psychologist and physiotherapist use was associated with 2yFAs and pFAs (Table 4). The OR increases over years when frequent attendance continues especially with regard to physiotherapist, psychologist, and specialist consultations.

**Table 4:** Factors associated with frequent attendance in multinomial logistic regression (n = 66,831)

	1-year-FA (2014–2016)			2-year-FA (2014–2016)			pFA (2014–2016)		
Factor	*n*	OR	95% CI	*n*	OR	95% CI	*n*	OR	95% CI
Professionals visited									
Physician	6513			1603			592		
Nurse	4119	3.43	3.25–3.63	1192	5.39	4.80–6.06	460	6.19	5.07–7.56
Physiotherapist	2932	4.73	4.48–5.00	1023	9.59	8.62–10.7	425	13.15	10.95–15.79
Psychologist	1174	6.19	5.71–6.70	467	11.92	10.6–13.5	196	14.44	11.99–17.40
Specialist consultation	1851	3.40	3.20–3.62	709	7.61	6.84–8.47	348	14.64	12.31–17.40
ICD-10									
M00-M99 Diseases of the musculoskeletal system and connective tissue	4796	4.59	4.33–4.86	1422	12.58	10.8–14.7	559	26.85	18.9–38.2
J00-J99 Diseases of the respiratory system	4254	2.88	2.73–3.05	1321	7.50	6.57–8.55	536	15.55	11.79–20.52
R00-R99 Symptoms, signs and abnormal clinical and laboratory findings, not elsewhere classified	2309	2.91	2.75–3.08	857	6.13	5.55–6.79	401	11.15	9.36–13.29
S00-T98 Injury, poisoning and certain other consequences of external causes	2198	2.87	2.71–3.03	792	5.68	5.13–6.30	349	8.58	7.25–10.15
F00-F99 Mental and behavioural disorders	1595	3.67	3.44–3.92	609	7.05	6.33–7.85	270	9.68	8.19–11.44
L00-L99 Diseases of the skin and subcutaneous tissue	1335	2.32	2.17–2.48	510	4.15	3.72–4.63	220	5.21	4.39–6.18
A00-B99 Certain infectious and parasitic diseases	1102	2.37	2.21–2.55	425	4.34	3.86–4.88	228	7.70	6.49–9.13
I00-I99 Diseases of the circulatory system	1129	2.13	1.98–2.29	403	3.38	2.99–3.81	168	4.00	3.32–4.83
G00-G99 Diseases of the nervous system	976	3.03	2.80–3.27	403	5.69	5.05–6.42	220	10.00	8.41–11.89
K00-K93 Diseases of the digestive system	934	2.60	2.40–2.81	379	4.75	4.20–5.36	202	7.93	6.65–9.44

ICD-10 = International Classification of Diseases

OR = Odds ratio (adjusted by sex, age, company size and field of industry), CI = Confidence interval

1.0 = reference group (non-FA = non-frequent attenders, patients that were never in the top decile were considered as a reference group)

FA status was defined as the top decile of attenders (frequent attender 10%, FA10),

1-year-FA = Patients that were in the top decile of attenders in one of the study years (2014, 2015 or 2016)

2-year-FA = Patients that were in the top decile in any two study years (2014, 2015 or 2016)

pFA = Patients that were in the top decile in all three study years (2014, 2015 and 2016)

In the table are presented the 10 largest ICD-10 groups

## Discussion

Nearly one in five frequent attenders in 2014 continued frequent use of services for the following two years. Persistent FAs are frequently women and employed in medium and large enterprises. Musculoskeletal disorders are more closely associated with pFA than other diagnostic groups. The association with mental disorders weakens as frequent attendance continues. The reasons for this effect should be examined further.

This study verifies in Finnish OH primary care environment that persistent frequent attenders create proportionally the most demand for the health care unit as previously seen in general practice (GP) setting [3]. The use of services and in particular physician consultations is substantial compared to non-FAs and also 1yFAs and 2yFAs. The pFA group of 592 patients made 23,797 visits to their primary care unit during the three study years. Given the cost of a physician visit compared to visits to other health care professionals, the economic effect created by this small group is notable. In our study nearly one out of five (19%) of FAs in 2014 continued as persistent frequent attenders, which is slightly more than in a Dutch study in general practice setting [3]. While the group of pFAs constituted 0.9% of the study population, they made 6% of all visits in the three study years. The three frequent attender groups (pFA, 2yFA and 1yFA) made up in total 40% of all consultations.

Our study is the first to describe how the use of other healthcare professionals varies between occasional and persistent frequent attenders. Visits to physiotherapists and psychologists were associated with persisting frequent attendance in particular and having consulted either them or a specialist increases the OR of belonging to pFA to almost 15. In this study we described how frequent attenders consult other healthcare professionals. It appears that although the use of physiotherapists and psychologists increases with pFAs, the dominance of physicians’ appointments is marked. Previously, in a GP setting specialist consultations have been linked to frequent attendance and use of multiple healthcare services to multimorbidity [7, 23]. Our study verifies the association of specialist consultation and frequent attendance and specifies the association with particularly persisting frequent attendance.

The significance of musculoskeletal disorders accumulates towards persisting frequent attendance. If diagnosed with a musculoskeletal disorder, the OR for being a pFA are over 26-fold (when adjusted for age, sex, employee size and industry). Although the association of musculoskeletal disorders and frequent attendance has been noted previously [18, 24, 25] its significance seems emphasized in the working population. Previous studies noted that musculoskeletal disorders are associated with visits to OH physicians and are one of the main work-related reasons for healthcare consultations [26, 27], which might explain this result in OH primary care. This result suggests that among the working age population diseases of the musculoskeletal system can be a more important factor driving frequent attendance than in the general practice setting. This is an observation that should be taken into account when planning identification and intervention strategies for frequent attenders in this context.

Our findings suggest that in particular those frequent attenders diagnosed with musculoskeletal disorders should be identified early. A follow up plan should be prepared, where a multiprofessional approach could be used in the spirit of Good Occupational Health Practice and the Occupational Health Care Act [28]. The accumulating pressure and weight on the system from frequent attendance is significant and cost-savings might be obtained if utilization could be increasingly planned and managed. Deeper analysis behind reasons for attendance [29] could be acquired through collaboration with other health care professionals.

OHS has close contact with the employers allowing, with the consent of the employee, also workplace interventions if seen necessary [30]. Although the likelihood of OH collaborative negotiation increases as the frequent use of services continues, these negotiations have been held for only 23% of pFA. Further studies should investigate if having attended an OH collaborative negotiation affects future frequent attendance. Interventions aimed at frequent attendance have shown encouraging results when subgroups such as depressed patients are targeted or a detailed analysis of reasons for attendance are carried out [29, 31]. If work related symptoms and performance difficulties cause visits to OH unit, workplace interventions, including OH collaborative negotiations, might be an effective way to address medically unsolvable reasons for attendance.

The association with mental and behavioural disorders also grows as frequent attendance persists, but diseases of the respiratory and nervous system show higher odds in association with pFA. An Estonian study found that depressed patients did not consult a physician significantly more than others when the follow up period was three years [32]. Effective recovery could explain this also in our study. However as mental disorders are one of the most common reasons for disability pensions, this issue should be studied further. It is not known if frequent attenders receive more disability pensions for mental disorders than others, which could also cause mental health diagnoses being less significant in the pFA group. Also in Finland, mental and behavioural disorders can also be treated in mental health services and units of secondary care. If a mental disorder persists, patients are often referred to these units. This might be one factor explaining why mental disorders appear less significant with pFA group. Similarly to Australian and Dutch primary care studies we found that persistent frequent attendance was associated with depression, but on the other hand we did not find an association with diabetes or heart problems [3, 5]. This might be due to our study material comprising of solely a working age population, some of whom may consult public practitioners for chronic diseases [26, 33]. The OH primary care setting most likely emphasizes the problems and illnesses affecting working ability [14].

The findings also indicate that respiratory diseases and diseases of the nervous system are closely associated with persistent high use of services in the working age population. An association of persistent high use of services with respiratory diseases has previously been reported in a primary care setting [3] and diseases of the nervous system have been associated with frequent attendance, but this confirms the connection also in persistent frequent attendance [25]. In turn, the high OR for the ICD R-group can be seen as indicative of medically unexplained physical symptoms (MUPS). The association of MUPS with persistent frequent attendance has been seen also in general practice setting [3] and is of importance as also medically unexplained symptoms increase the risk of long-term sickness absence [34]. The finding that injuries have higher odds for persistent FA is interesting, and might reinforce the perception that persistent frequent attenders are more vulnerable as also indicated in a previous study [35]. Multimorbidity is associated with frequent attendance and appears to increase as frequent attendance persists, as also seen previously [3]. As a whole, no single factor differentiates these groups from each other but rather, these factors seem to exist on a continuum.

Our study has certain limitations. Our study population differs from the general practice setting to some extent in terms of patient age and working status, and we assume that these demographic differences possibly accentuate different factors than what would rise in general practice setting. The lack of occupational status and education are limitations to the study as these are not available in medical records. Human error may be present when using medical record data, but the large sample likely dilutes the effect. Retrospective study sets limitations to variables used, which are also limited by what is and can be registered in the electronic patient registers.

On the other hand our data allow a unique perspective to this particular group given our nationwide material covering largely different service sectors and both rural and urban areas with employees with variety of employment lengths and industries. The distribution of employers’ size and industry resembles the general distribution of employers according to Statistics Finland [36]. The equal age distribution within the working age population and equal gender distribution, allows generalization outside this particular context. Strengths of the study are large sample and longitudinal study design allowing for interpretation of predictive factors of persistent frequent attendance. The health care records in Finland are accurate and comprehensive allowing for good quality data. For example, the ICD-10 classified diagnostic code was missing in only 1% of the visits. In this study we did not have access to use of other health care services, but a previous study indicates that when OHS primary care is available it is often used as sole primary care provider [26].

## Conclusions

Diseases of the musculoskeletal system are emphasized among persistent frequent attenders of occupational health primary care. This could be explained by the demands of working life or that the conditions are work-related. As it seems that persistent frequent attenders create the most demand for their primary care unit, it is necessary to further examine whether they are also at risk of disability and sickness absences. When planning future interventions aimed at frequent attenders, the subgroup suffering from musculoskeletal disorders should be considered. Among the working age patients, identified disorders’ work-relatedness should be considered.

## Abbreviations

CI: Confidence interval
FA10: Frequent attender 10% (patients in the top decile of annual visits to healthcare professionals)
GP: General practice
MUPS: Medically unexplained physical symptoms
OH: Occupational health
OHS: Occupational health services
OR: Odds ratios
